# Effectiveness of electrical stimulation for postoperative pain in patients with osteosarcoma: A systematic review protocol of clinical controlled trial

**DOI:** 10.1097/MD.0000000000016783

**Published:** 2019-08-09

**Authors:** Tao Yu, Hua-yu Tang, Tian-shu Wang, Wei Wei

**Affiliations:** Second Ward of Orthopedis Department, First Affiliated Hospital of Jiamusi University, Jiamusi, China.

**Keywords:** effectiveness, electrical stimulation, osteosarcoma, postoperative pain, safety

## Abstract

**Background:**

This study aims to investigate the effectiveness and safety of electrical stimulation (ES) for postoperative pain (PPP) in patients with osteosarcoma systematically.

**Methods:**

We will systematically search the following electronic databases from inception to the May 1, 2019: MEDILINE, Cochrane Library, EMBASE, Web of Science, Springer, and CNKI without language restrictions. All literatures of randomized controlled trials (RCTs) and case-controlled studies (CCSs) of ES for PPP in patients with osteosarcoma will be included. RevMan 5.3 software (Cochrane Community; London, UK) and STATA 15.0 software (StataCorp; College Station) will be used for statistical analysis. Cochrane risk of bias will be used for methodological quality assessment for RCTs and Newcastle-Ottawa Scale will be utilized for CCSs.

**Results:**

This study will assess the clinical effectiveness and safety of ES for PPP in patients with osteosarcoma through assessing primary outcome of pain intensity and secondary outcomes of frequency of rescue analgesic use, cumulative morphine consumption, quality of recovery, as well as adverse events.

**Conclusion:**

This study will provide latest evidence on effectiveness and safety of ES for PPP in patients with osteosarcoma, and may also provide guidance for both clinician and further studies.

**Dissemination and ethics:**

This study does not require ethical approval, because it will not analyze the individual patient data. Its results are expected to be published in peer-reviewed journals.

**Systematic review registration:**

PROSPERO CRD42019135790.

## 1 Introduction

Osteosarcoma is the most common type of bone cancer in children and teenagers.^[1–3]^ Previous studies have reported that the incidence rate of osteosarcoma is 4 to 5 patients per 1,000,000 persons.^[4,5]^ Other studies have reported that its 5-year overall survival rate and 5-year disease-free survival rate are about 50% to 60% and 40%, respectively.^[6–8]^ The huge amount cost of treatment and care also brings heavy burden for both families and society.^[9,10]^

Currently, although a variety of therapies for this condition has been reported, the efficacy is still not satisfied.^[11–13]^ These therapies mainly include medications and surgery.^[14,15]^ Though many patients choose surgery for their treatment, it also accompanies a lot of complications, such as postoperative pain (PPP), infection, and so on.^[16,17]^

Electrical stimulation (ES) has been reported to treat a variety of pain conditions effectively and safety.^[18–23]^ Furthermore, many studies also reported to use ES for the treatment of PPP in patients with osteosarcoma.^[24–26]^ However, no study has systematically assessed its effectiveness and safety for PPP. Thus, this study will investigate the effectiveness and safety of ES for osteosarcoma patients with PPP.

## 2 Methods

### 2.1 Objective

The aim of this study is to investigate the effectiveness and safety of ES for osteosarcoma in patients with PPP.

### 2.2 Study registration

We have registered this study on http://www.crd.york.ac.uk/ PROSPERO with CRD42019135790. It has been designed and reported according to the guidelines of the Preferred Reporting Items for Systematic Reviews and Meta-Analysis (PRISMA) Protocol statement.^[27]^

### 2.3 Inclusion criteria for study selection

#### 2.3.1 Type of study

We will include all randomized controlled trials (RCTs) and case-controlled studies (CCSs) of ES for PPP in patients with osteosarcoma. However, animal studies, reviews, case studies, and non-case CCSs will all be excluded.

#### 2.3.2 Type of participants

All osteosarcoma participants with PPP will be included without any limitations of age, sex, and race.

#### 2.3.3 Type of interventions

The patients in experimental group must be treated with any forms of ES, such as electrical muscle stimulation, Russian ES, neuromuscular ES, functional ES, and transcutaneous electrical nerve stimulation.

The participants in the control group have been treated with any non-ES therapies.

#### 2.3.4 Type of outcome measurements

The primary outcome of pain intensity can be measured by Numerical Rating Scale, or any other pain scales. The secondary outcomes can be measured by the frequency of rescue analgesic use, cumulative morphine consumption, and quality of recovery. The dose, frequency of all analgesic will be monitored and recorded during the period of hospital stay. The cumulative morphine consumption will also be documented during the period of hospital stay. The quality of recovery will be measured by the Quality of Recovery-9 or other relevant scales, which is used to evaluate the patient's quality of recovery after anesthesia. In addition, any adverse events will also be assessed.

### 2.4 Search methods for the identification of studies

#### 2.4.1 Electronic searches

The following databases will be systematically searched from inception to the May 1, 2019: MEDILINE, Cochrane Library, EMBASE, Web of Science, Springer, and CNKI without language restrictions. The search details for MEDLINE are demonstrated in Table 1. The equivalent strategies will be used to any other electronic databases.

**Table 1:**
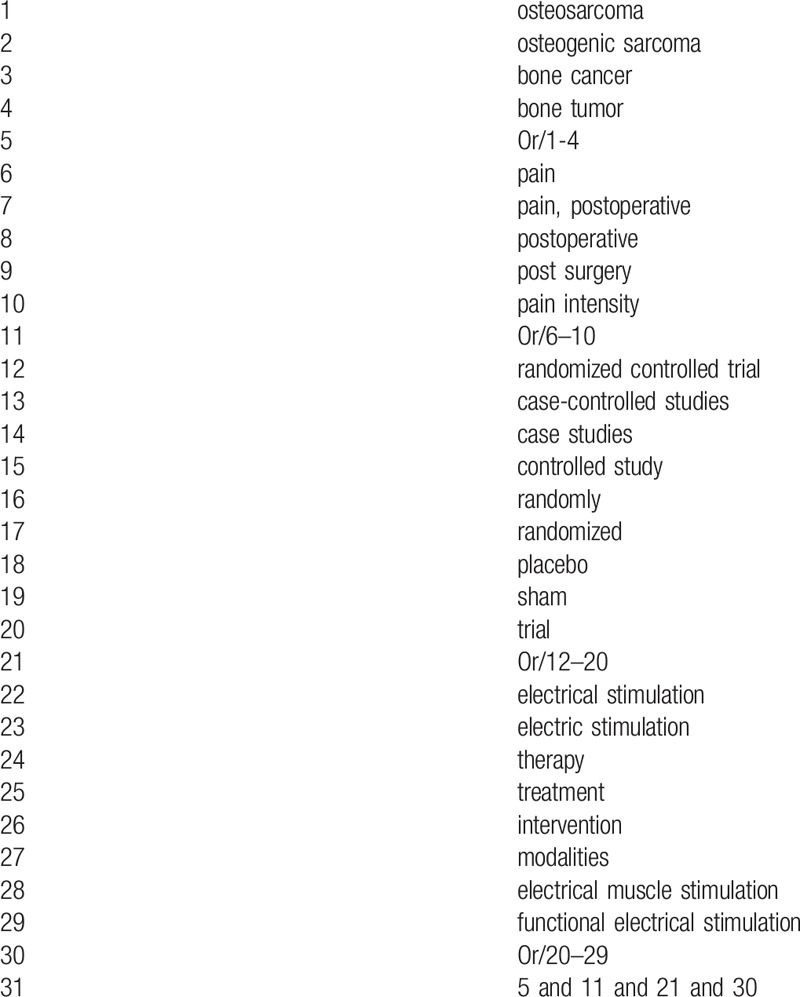
Search strategy for MEDLINE database.

#### 2.4.2 Search for other resources

In addition, any clinical registry websites, dissertations, and reference lists of relevant reviews will be searched to avoid missing any potential studies.

### 2.5 Data collection and analysis

#### 2.5.1 Study selection

Two reviewers will independently operate all literature selection according to the predefined study selection eligibility. In case of any disagreements regarding the study selection between 2 reviewers, a third reviewer will take part in to help solve them by discussion. There are 2 stages for all literature selection. At first stage, the titles and abstracts of all literatures will be checked, and irrelevant literatures will be excluded. At the second stage, all remaining literatures will be read by full-text to further determine if they meet all eligibility criteria. The process of 2-stage study selection will be presented in the PRISMA flow chart.

#### 2.5.2 Data extraction and management

Two reviewers will independently carry out data extraction according to the PRISMA flowchart and pre-designed eligibility criteria. The extracted information comprises of title, authors, year of publication, location, study design, sample size, study methods, intervention details, outcome measurements, and adverse events. Any divergences between 2 reviewers will be settled down by a third reviewer through discussion.

#### 2.5.3 Missing data management

For any insufficient or missing, or unclear data, we will contact original corresponding authors to obtain them. If we can not achieve them, only available data will be analyzed in this study.

#### 2.5.4 Methodological quality assessment

Two reviewers will independently assess the methodological quality for each eligible study. Any disagreements between 2 reviewers will be resolved by a third reviewer through discussion. For RCTs, their methodological quality will be measured by Cochrane risk of bias tool. For CCSs, their methodological quality will be assessed by Newcastle-Ottawa Scale.

### 2.6 Statistical analysis

#### 2.6.1 Measurement of treatment effect

The continuous data will be shown with mean difference or standardized mean difference and 95% confidence intervals (CIs). The dichotomous data will be performed with odd ratio or risk ratio and 95% CIs.

#### 2.6.2 Assessment of heterogeneity

The test of *I*^2^ will be utilized to detect the heterogeneity among eligible studies. A fair heterogeneity is defined as *I*^2^ ≤ 50%. On the other hand, significant heterogeneity is defined as *I*^*2*^ > 50%.

#### 2.6.3 Data synthesis

If *I*^2^ ≤ 50%, the outcome data will be synthesized by a fixed-effect model. In addition, we will also carry out meta-analysis if it is possible. Otherwise, if *I*^*2*^ > 50%, the outcome data will be synthesized by a random-effect model. At the same time, we will also conduct subgroup analysis to find any factors that may lead to high heterogeneity. If significant heterogeneity is still detected after subgroup analysis, outcome data will not be synthesized, and meta-analysis will not be performed. Instead, we will report outcome results using narrative summary description.

#### 2.6.4 Subgroup analysis

Subgroup analysis will be carried out according to the different forms of treatments, controls, and outcomes.

#### 2.6.5 Sensitivity analysis

We will carry out sensitivity analysis to check the robustness of the pooled results based on the different methodological quality and statistical models.

#### 2.6.6 Publication bias

If this study includes >10 eligible studies, we will carry out funnel plot^[28]^ and Egger test to check if there is publication bias in this study.^[29]^

## 3 Discussion

PPP is gravely tormenting patients and greatly reduces their quality of life. A variety of clinical studies have reported ES can help to treat PPP in patients with osteosarcoma.^[24–26]^ Thus, in this study, we will comprehensively search more potential literatures and will systematically evaluate the effectiveness and safety of ES for osteosarcoma patients with PPP. The results of this study will summarize the updated evidence on the effectiveness and safety of ES for osteosarcoma patients with PPP. It may also provide beneficial evidence for the clinical practice and health policy-makers.

## Author contributions

**Conceptualization:** Hua-yu Tang, Tian-shu Wang, Wei Wei.

**Data curation:** Tao Yu, Hua-yu Tang, Wei Wei.

**Formal analysis:** Tao Yu, Hua-yu Tang, Tian-shu Wang.

**Funding acquisition:** Wei Wei.

**Investigation:** Wei Wei.

**Methodology:** Tao Yu, Hua-yu Tang, Tian-shu Wang.

**Project administration:** Wei Wei.

**Resources:** Tao Yu, Hua-yu Tang, Tian-shu Wang.

**Software:** Tao Yu, Hua-yu Tang, Tian-shu Wang.

**Supervision:** Wei Wei.

**Validation:** Tao Yu, Wei Wei.

**Visualization:** Hua-yu Tang, Tian-shu Wang, Wei Wei.

**Writing – original draft:** Tao Yu, Hua-yu Tang, Tian-shu Wang, Wei Wei.

**Writing – review & editing:** Tao Yu, Hua-yu Tang, Tian-shu Wang, Wei Wei.
